# Septic pulmonary embolism in Somali children: a retrospective study from a tertiary hospital

**DOI:** 10.3389/fped.2026.1804752

**Published:** 2026-05-13

**Authors:** Mohamed Nur Ali, Mustafa Gahnug, Ali Abdi Jama, Mahad Sadik Mukhtar, Mohamed Yaqub Hassan, Farah Ali Ahmed, Yasir Khalif Ali, Farah Abdullahi Ismail, Shuayb Moallim Ali Jama, Nur Adam Mohamed, İbrahim Abukar Abdi, Abdisalam Ismail Hassan, Ismail Gedi Ibrahim, Abdirahman Mohamed Jimale, Mohamed Sheikh Hassan, Said Abdirahman Ahmed

**Affiliations:** 1 Department of Pediatric, Mogadishu Somali-Turkish Training and Research Hospital, Mogadishu, Somalia; 2 Faculty of Medicine & Surgery, Al Hayat Medical University, Mogadishu, Somalia; 3 Department of Pulmonology, Mogadishu Somali-Turkish Training and Research Hospital, Mogadishu, Somalia; 4 Department of Radiology, Mogadishu Somali-Turkish Training and Research Hospital, Mogadishu, Somalia; 5 Department of Psychiatry, Mogadishu Somali-Turkish Training and Research Hospital, Mogadishu, Somalia; 6 Department of Emergency Medicine, Mogadishu Somali-Turkish Training and Research Hospital, Mogadishu, Somalia; 7 Faculty of Medicine and Health Sciences, Jamhuriya University of Science and Technology, Mogadishu, Somalia; 8 Department of Public Health, Faculty of Health Science, University of Somalia (UNISO), Mogadishu, Somalia; 9 Department of Neurology, Mogadishu Somali-Turkish Training and Research Hospital, Mogadishu, Somalia; 10 Department of Cardiology, Mogadishu Somali-Turkish Training and Research Hospital, Mogadishu, Somalia

**Keywords:** children, septic pulmonary embolism, Somalia, Staphylococcus aureus, thrombophlebitis

## Abstract

**Background:**

Septic pulmonary embolism (SPE) is an under-recognized cause of severe infection in children, particularly in low-resource settings. Evidence from Somalia is absent, despite high rates of skin and soft-tissue infection and limited diagnostic capacity. This study described the clinical characteristics, microbiology, radiological features, management, and outcomes of pediatric SPE in a Somali tertiary hospital.

**Methods:**

We conducted a retrospective review of children aged < 18 years diagnosed with SPE between 2021 and 2025. Data on demographics, clinical presentation, laboratory parameters, radiological findings, microbial isolates, treatments, and outcomes were extracted. Continuous variables were summarized as mean ± SD or median (IQR) and categorical variables as counts and percentages. ICU and non-ICU groups were compared using *t*-tests or Mann–Whitney U tests and Fisher's exact test where appropriate. Effect sizes (Cohen's d, rank-biserial correlation) were calculated. Due to the small number of events and absence of mortality, no multivariable modelling was performed.

**Results:**

Fifteen children met diagnostic criteria for SPE; median age was 12 years and 80% were male. Skin and soft-tissue infections, trauma-related sepsis, and septic thrombophlebitis were the most common sources. *Staphylococcus aureus* (MSSA/MRSA) accounted for most culture-positive cases while coagulase-negative staphylococci and Gram-negative organisms were less frequent. Radiological findings included peripheral nodules, cavitation, and pleural disease. Five children (33%) required ICU admission and anticoagulation was used in 93%. ICU patients were younger, had higher inflammatory markers and experienced significantly longer hospital stays (*p* = 0.031; d = 1.49). No in-hospital deaths occurred.

**Conclusion:**

Pediatric SPE in Somalia is predominantly staphylococcal in origin and commonly arises from skin, soft-tissue, or trauma-related infections. Despite limited imaging and microbiology resources, characteristic radiological patterns and marked inflammation facilitate diagnosis. Younger age, higher leukocytosis, and prolonged hospitalization were associated with ICU admission, but overall outcomes were favorable with timely antimicrobial therapy, anticoagulation, and procedural interventions. These findings provide urgently needed baseline evidence for SPE in Somali children and underscore the importance of early recognition in resource-limited settings.

## Introduction

Septic pulmonary embolism (SPE) is the lodging of infected thrombi from a primary extra-pulmonary infectious focus into the pulmonary vasculature and represents a rare but potentially life-threatening entity in children. SPE remains under-recognized, often misdiagnosed and under-reported and with paediatric data particularly scarce **(**1, 2). Overall, paediatric venous thromboembolism (VTE), including pulmonary embolism (PE) is much less common than in adults (3, 4). However, recent evidence indicates a rising incidence of acute PE in children and adolescents. A nationwide United States analysis reported an annual incidence of approximately 3.5 PE-related hospitalisations per 100,000 children between 2016 and 2019 (5). Although this analysis did not differentiate septic from non-septic PE, it highlights that PE is no longer exceedingly rare in paediatrics. Increasing use of intravascular devices, prolonged survival of children with chronic diseases and more complex medical interventions may collectively predispose to SPE (5, 6).

Despite this overall trend, SPE itself remains substantially underreported. A retrospective analysis covering 2015–2022 documented only a small number of paediatric SPE cases, underscoring its rarity even in tertiary centres (2). In children, PE and by extension SPE is usually associated with identifiable clinical risk factors, more frequently than in adults (5, 6). Common predisposing conditions include central venous catheters, malignancy, recent surgery, congenital or acquired pro-thrombotic states, trauma and chronic illnesses (7–9). For SPE specifically, infected emboli typically originate from deep tissue infections such as osteomyelitis or septic arthritis, soft-tissue abscesses, septic thrombophlebitis, infected vascular lines or right-sided infective endocarditis (10, 11).

The pathophysiological process involves dislodgement of septic thrombi from a primary infectious focus, entry into the venous system, transit through the right heart and subsequent occlusion of pulmonary arterial branches. This leads to pulmonary infarction, necrosis, cavitation, abscess formation and intense inflammatory injury (12, 13).

Clinically, SPE in children presents with non-specific symptoms such as fever, cough, tachypnoea or dyspnoea, chest pain and occasionally hemoptysis — features that overlap with common paediatric respiratory infections (1, 13). As a result, diagnosis is often delayed or missed; some reported cases of SPE have only been confirmed after significant clinical deterioration or even post-mortem (4, 14).

Radiographic findings on chest radiography are typically non-specific, often showing patchy infiltrates or consolidation that may be interpreted as pneumonia (14). In contrast, contrast-enhanced computed tomography (CT) provides greater diagnostic sensitivity: typical findings include multiple peripheral nodules, wedge-shaped opacities, cavitary lesions and occasionally the characteristic “feeding-vessel sign,” which strongly suggests SPE (2, 12).

Microbiologically, gram-positive organisms—particularly *Staphylococcus aureus* (both methicillin-sensitive and methicillin-resistant) — are the predominant pathogens implicated in paediatric SPE (1, 15). However, unusual or opportunistic organisms have been reported, particularly among immunocompromised children or those with indwelling vascular devices (10, 12). Blood cultures may be negative in a substantial proportion of cases, especially in children who received antibiotics prior to sampling, complicating definitive microbiological diagnosis (15).

SPE in children is associated with significant morbidity and may be life-threatening. A 2023 retrospective paediatric study demonstrated that early diagnosis combined with timely, aggressive management — including appropriate antimicrobial therapy and effective drainage or surgical control of the primary infective focus — improves outcomes (2). Adult studies have reported mortality rates as high as 20%, although paediatric mortality data remain limited (15). Delays in diagnosis are common in settings with restricted access to CT imaging, vascular ultrasound, microbiology, or paediatric intensive care support — challenges that are magnified in low-resource environments (12, 14).

Despite increasing recognition of PE in children generally, there remains a marked paucity of data on SPE in paediatric populations, especially from low- and middle-income countries (LMICs). Most published literature originates from high-income regions, with only a few small paediatric case series available in recent years (1, 10). African data on SPE remain limited, despite evidence of high rates of *Staphylococcus aureus* bacteremia, prevalent head-and-neck infections progressing to septic thrombophlebitis, and a substantial burden of severe pediatric infections—often complicated by delayed presentation and limited critical care resources across the continent (16–18).

Musculoskeletal infections are highly prevalent across Africa, with Nigerian studies reporting frequent pediatric osteomyelitis complicated by abscess formation and bacteremia, while Kenyan data demonstrate a substantial burden of invasive bacterial infections associated with strong inflammatory responses and increased risk of embolic spread (19, 20). A decade-long study in Rwanda demonstrated persistently high rates of pediatric bloodstream infections with delayed recognition, while data from Qatar highlight bone and joint infections—commonly caused by *Staphylococcus aureus* and occasionally Gram-negative organisms such as *Brucella* spp.—as important sources of septic emboli (21, 22). Procedural and device-related factors contribute significantly, as evidenced by high rates of central venous catheter–associated infection and thrombosis in Ethiopian pediatric oncology units, alongside broader diagnostic limitations and trauma-related catheter use in resource-limited African settings, all of which may promote septic thrombus formation and embolization (23–25).

To our knowledge, no pediatric case series of septic pulmonary embolism (SPE) have been reported from the Horn of Africa, with only limited data from sub-Saharan Africa. In Somalia, restricted access to advanced imaging, microbiological diagnostics, and pediatric intensive care may contribute to delayed diagnosis and management. This study retrospectively described the clinical, radiological, microbiological, and management characteristics of pediatric SPE in a tertiary hospital, providing hypothesis-generating data to inform future research in resource-limited settings.

## Methodology

### Study design and setting

We conducted a retrospective observational study of children admitted with septic pulmonary embolism (SPE) to a tertiary referral hospital in Somalia. The study included all eligible admissions to the pediatric medical and surgical wards and the pediatric intensive care unit (PICU) between 2021 and 2025.

### Patient selection

All consecutive patients aged < 18 years with a diagnosis of SPE during the study period were screened for inclusion. SPE was diagnosed by the treating team based on a compatible clinical presentation and characteristic radiological features on chest computed tomography (CT) and/or chest radiography, in the context of a proven or strongly suspected extrapulmonary infectious focus.

Due to limited availability of advanced imaging, not all patients underwent Chest CT; in such cases, diagnosis was based on a combination of clinical presentation and chest radiographic findings.

**Inclusion criteria were**:

Age < 18 years at admission.

Radiological findings suggestive of septic emboli (e.g., multiple nodular or cavitary lesions, peripheral or wedge-shaped opacities, “feeding vessel” sign).

Identifiable or suspected extrapulmonary infectious focus (e.g., skin/soft-tissue infection, osteomyelitis, septic thrombophlebitis, otitis media, pneumonia/empyema) and/or positive blood or site culture supporting infection.

**Patients were excluded if**:

An alternative non-infective explanation for the pulmonary lesions was documented (e.g., malignancy, non-septic thromboembolism).

Clinical and imaging data were insufficient to confirm SPE.

### Data collection and variables

Data were abstracted from paper and electronic medical records using a structured case-report form. The following variables were collected:

Demographics and baseline characteristics: age, sex, chronic comorbidities (cardiovascular disease, chronic kidney disease, liver disease, neurologic disease), presence of bronchiectasis, history of nosocomial infection, venous catheter infection, steroid use, and infective endocarditis.

Predisposing factors and septic foci: trauma or snake bite, soft-tissue and bone infection, cellulitis, abscess, septic arthritis, empyema, otitis media, and “focus not identified”.

Clinical presentation at admission: fever, cough, shortness of breath, chest pain, hemoptysis, sore throat, myalgia, hypotension and need for PICU admission.

Laboratory investigations: white blood cell (WBC) count, neutrophil and lymphocyte counts, hemoglobin, platelet count, C-reactive protein (CRP), erythrocyte sedimentation rate (ESR), serum creatinine, blood urea nitrogen/urea, liver transaminases (AST, ALT), serum albumin (if available), international normalized ratio (INR), D-dimer, and arterial partial pressure of oxygen (PaO₂) where measured.

**Radiological findings:** presence, distribution and type of pulmonary lesions on CT or chest radiograph (cavitary, nodular, ground-glass opacities, consolidation, feeding-vessel sign), pleural effusion/empyema, and evidence of extrapulmonary thrombosis on Doppler ultrasound or echocardiography when performed.

**Outcomes:** admission to PICU, in-hospital complications (e.g., empyema, respiratory failure, extension of thrombus, bleeding events) and length of hospital stay (LOS, days). No in-hospital deaths occurred in this cohort.

Missing data were recorded as such and no imputation was performed; analyses were conducted on a complete-case basis for each variable.

### Definitions

Septic pulmonary embolism was defined as radiological evidence of multiple peripheral nodular, cavitary, or wedge-shaped infiltrates compatible with embolic infection in the presence of a primary infectious focus or documented bacteremia.

Complications were defined as any new clinically significant event during admission attributable to SPE or its treatment (e.g., empyema requiring drainage, respiratory failure requiring mechanical ventilation, bleeding related to anticoagulation, or extension of thrombosis).

ICU admission was defined as any stay in the pediatric intensive care unit at any point during the hospitalization.

### Statistical analysis

Given the small sample size, the analysis was primarily descriptive and exploratory. Continuous variables were summarized as mean ± standard deviation (SD) for approximately normally distributed data and as median and interquartile range (IQR) for skewed distributions. Normality was assessed visually using histograms and, where appropriate, the Shapiro–Wilk test. Categorical variables were presented as counts and percentages.

For exploratory between-group comparisons, we examined:

ICU vs. non-ICU admissions, and Presence vs. absence of in-hospital complications.

For continuous variables, we used Student's *t*-test when normality assumptions were reasonable and Mann–Whitney U test otherwise. For categorical variables, we used Fisher's exact test (or chi-square test when expected cell counts were adequate).

Given the very limited number of outcome events and the absence of in-hospital mortality, no multivariable regression or survival (Cox) modelling was performed. All *p*-values were two-sided, and a *p*-value < 0.05 was considered statistically significant. Statistical analyses were performed using standard statistical software (SPSS).

Ethical approval was obtained from the Mogadishu Somali Türkiye Training and Research Hospital's institutional review board MSTH/22677 reference number available upon request. The study adhered to the principles of the Declaration of Helsinki and local ethical guidelines. Informed consent was obtained from each patient's legal guardian.

## Results

### Baseline characteristics

Fifteen children with confirmed septic pulmonary embolism were included (Table 1). The median age was 12 years (IQR: 9.5–14), and 12/15 (80%) were male. All patients were previously healthy with no chronic cardiovascular, renal, hepatic, or neurologic diseases documented. Bronchiectasis was present in five patients (33%), while four patients (27%) presented with trauma or snake bite as a predisposing factor. In cases involving snake bite, this was considered a potential primary inoculation site leading to subsequent soft-tissue infection and septic embolization. A septic focus was identified in most cases, most commonly cellulitis (40%), soft-tissue infection or abscesses (∼50% combined), and less frequently septic arthritis or empyema. Blood cultures or site cultures were performed in 14 children; 9/15 (60%) had microbiologically confirmed infection—predominantly *Staphylococcus aureus* (MSSA/MRSA) and coagulase-negative staphylococci.

**Table 1:** Baseline characteristics of children with septic pulmonary embolism (*n* = 15).

Characteristic	Value
Age (years), mean ± SD	11.5 ± 4.2
Age (years), median (IQR)	12.0 (9.5–14.0)
Sex, male	12/15 (80.0%)
Bronchiectasis	5/15 (33.3%)
Trauma/Injury	4/15 (26.7%)
Nosocomial infection	1/15 (6.7%)
ICU admission	5/15 (33.3%)

### Clinical presentation and laboratory findings

Fever was nearly universal (93%), followed by cough (60%) and shortness of breath (53%). Myalgia was present in 73% and chest pain in 40%. No patient had documented hypotension on admission.

**Figure 1:**
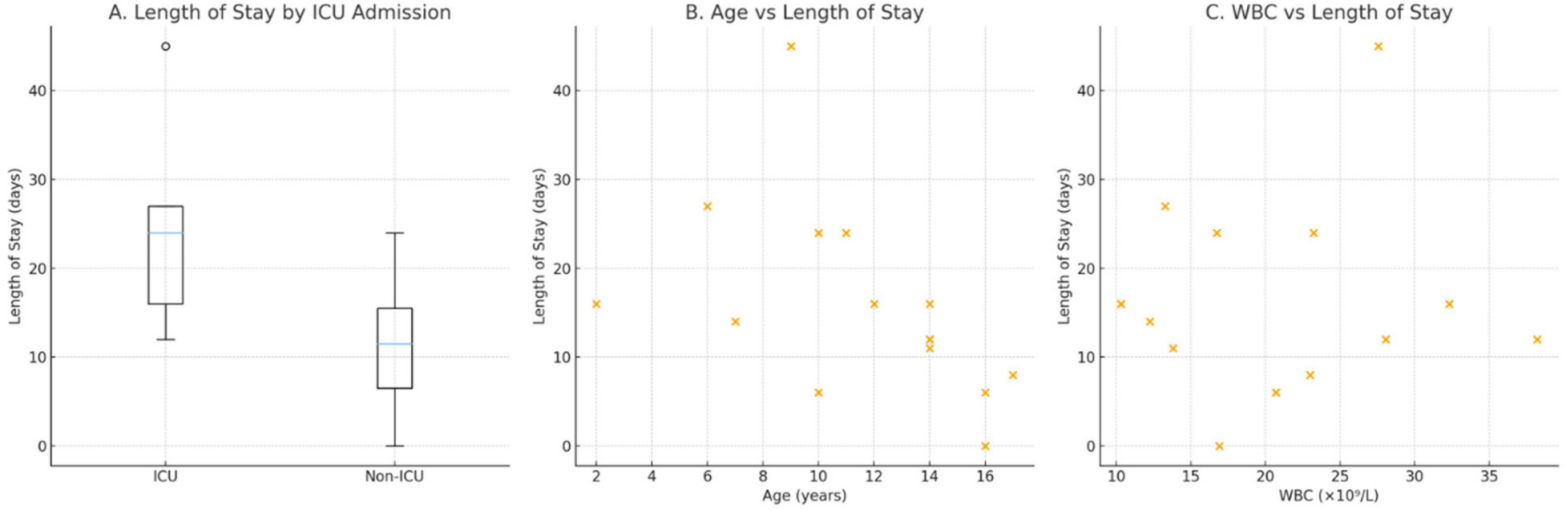
Clinical severity indicators in Somali children with septic pulmonary embolism. **(A)** Length of hospital stay by ICU versus non-ICU admission. **(B)** Relationship between age and length of stay. **(C)** Association between white blood cell (WBC) count and length of stay.

Laboratory findings demonstrated marked systemic inflammation, with a mean WBC count of 20.5 × 10⁹/L, neutrophilia, and markedly elevated CRP (median ∼220 mg/L) (Table 2, Figure 1).

**Table 2:** Laboratory parameters at admission in children with septic pulmonary embolism, including measures of central tendency and dispersion.

Variable	n	Mean	SD	Median	IQR
WBC ( × 10⁹/L)	15	20.49	8.34	20.69	13.53–25.39
Neutrophils	15	17.27	7.15	18.71	12.14–20.51
Platelets ( × 10⁹/L)	15	300.93	219.28	266.00	149.0–408.5
CRP (mg/L)	14	221.00	107.52	223.50	118.5–319.0
ESR (mm/h)	5	58.40	18.69	56.00	51.0–70.0
Creatinine (mg/dL)	12	0.48	0.24	0.45	0.27–0.58
Urea (mg/dL)	15	39.53	19.99	41.00	20.0–53.0
AST (U/L)	15	75.87	56.31	59.00	41.5–82.0
ALT (U/L)	15	37.80	24.30	32.00	20.5–43.5
INR	13	1.86	1.46	1.44	1.13–1.74
D-Dimer	4	6.87	2.57	6.79	5.01–8.65
PAO₂ (mmHg)	7	80.86	75.07	61.00	46.0–68.0

CRP values were measured and reported in mg/L.

WBC, white blood cell count; CRP, C-reactive protein; INR, international normalized ratio; ESR, erythrocyte sedimentation rate.

Mild to moderate anemia was common (mean hemoglobin 9.3 g/dL). Coagulation abnormalities were observed, with a mean INR of 1.86, and D-dimer was markedly raised in all tested children. Serum albumin levels, available in a subset, were low. Transaminases were mildly to moderately elevated.

### Radiological and microbiological features

Pulmonary imaging (CT or CXR) demonstrated typical SPE patterns, including peripheral nodules, cavitary lesions, wedge-shaped infiltrates, and ground-glass opacities. Several children had pleural effusions or empyema requiring drainage. Doppler ultrasound and/or echocardiography revealed evidence of venous thrombosis in a subset, consistent with the high rate of anticoagulant therapy.

Microbiological confirmation was obtained in 9 patients (60%). Staphylococcal species accounted for the majority of isolates, including MSSA, MRSA, and coagulase-negative staphylococci (Table 3). One isolate of *Acinetobacter calcoaceticus* and one *Streptococcus* species were identified.

**Table 3:** Microbiological profile of pathogens identified in children with septic pulmonary embolism.

Organism	*n*
Negative culture	5
Coagulase-negative staphylococci (CoNS)	3
Methicillin-susceptible *Staphylococcus aureus* (MSSA)	**3**
Methicillin-resistant *Staphylococcus aureus* (MRSA)	1
*Streptococcus* spp.	1
*Acinetobacter calcoaceticus*	1
No culture performed	1

Bold values indicate statistically significant results (*p* < 0.05).

### Treatment interventions

Broad-spectrum antimicrobials were used in all patients. The most frequently administered antibiotics were meropenem (73%) and vancomycin (73%), followed by clindamycin, piperacillin-tazobactam, linezolid, ceftriaxone, amikacin, and others depending on clinical course and culture results.

Systemic anticoagulation was administered in 14 patients (93%), reflecting the embolic and thrombotic phenotype of the cohort. Oxygen therapy was required in 5 patients (33%), and 3 patients (20%) underwent chest tube insertion. Abscess drainage was performed in 40%.

### ICU admission and complications

Five children (33%) required admission to the intensive care unit. ICU patients were younger (mean 8.4 vs. 13.0 years) and had longer hospital stays (mean 24.8 vs. 11.3 days) than non-ICU patients. They also had higher inflammatory markers, including WBC and neutrophil counts.

In-hospital complications occurred in 5/15 patients (33%), including empyema, persistent respiratory distress, or thrombus progression (Table 4). No cases of anticoagulation-related major bleeding were reported.

**Table 4:** Antimicrobial therapies and supportive interventions administered to children with septic pulmonary embolism.

Intervention	*n*/*N* (%)
Meropenem	11/15 (73.3%)
Vancomycin	11/15 (73.3%)
Piperacillin–tazobactam	3/15 (20.0%)
Linezolid	1/15 (6.7%)
Ceftriaxone	2/15 (13.3%)
Amikacin	1/15 (6.7%)
Systemic anticoagulant use	14/15 (93.3%)
Oxygen therapy	5/15 (33.3%)
Chest tube insertion	3/15 (20.0%)
Drainage of skin abscess	6/15 (40.0%)

### Between-group comparisons

Exploratory comparisons were performed between ICU and non-ICU patients and between those with and without complications. ICU patients had significantly longer hospital stays and tended to have higher inflammatory markers, although statistical significance was limited by sample size. Detailed comparisons between ICU and non-ICU patients are presented in Table 5.

**Table 5:** Comparison of ICU vs. non-ICU patients with *p*-values and effect sizes.

Variable	Group	n	Mean	SD	Median	IQR	Mann–Whitney *p*	Effect size (d/r_rb)
Age (years)	ICU	5	8.40	4.62	9.00	6.0–11.0	0.063	**d** **=** **–1.25/r** **=** **+** **0.62**
	Non-ICU	10	13.00	3.20	14.00	10.5–15.5		
WBC ( × 10⁹/L)	ICU	5	24.88	7.25	27.56	23.22–28.05	0.098	**d** **=** **+** **0.83/r** **=** **–0.56**
	Non-ICU	10	18.29	8.30	16.82	12.64–20.69		
CRP (mg/L)	ICU	4	215.25	104.93	218.00	131.5–301.75	0.832	**d** **=** **–0.07/r** **=** **+** **0.10**
	Non-ICU	10	223.30	114.05	223.50	128.99–321.0		
Hospital stay (days)	ICU	5	24.80	12.79	24.00	16.0–27.0	**0**.**031**	**d** **=** **+** **1.49/r** **=** **–0.72**
	Non-ICU	10	11.30	6.73	11.50	6.5–15.5		

WBC, white blood cell count; CRP, C-reactive protein; ICU, intensive care unit; SD, standard deviation; IQR, interquartile range.

Bold values indicate statistically significant results (*p* < 0.05).

Effect sizes were calculated using Cohen's d for continuous variables and rank-biserial correlation for Mann–Whitney U tests. Large effect sizes were observed for age (d = –1.25) and WBC count (d = 0.83), indicating substantial clinical differences between ICU and non-ICU patients. Length of stay demonstrated a very large effect (d = 1.49, r_rb = –0.72), consistent with the statistically significant difference (*p* = 0.031). CRP levels showed negligible effect size (d = –0.07).

Complications were more common in patients with high CRP, high INR, and marked leukocytosis. Fisher's exact test was used for categorical comparisons, and Mann–Whitney U or Student's *t*-test for continuous variables, depending on distribution.

#### Outcomes

The median length of hospital stay was 14 days (IQR: 12–18), with a range of 0–45 days. All patients survived to hospital discharge, and no in-hospital mortality was recorded.

## Discussion

In this retrospective series of Fifteen Somali children with septic pulmonary embolism, we observed a characteristic pattern of disease involving previously healthy school-aged children, with a predominance of skin and soft-tissue infections, *Staphylococcus aureus* bacteremia, and thrombotic complications. Our findings align with the limited African literature describing pediatric SPE, but also reveal important contextual differences relevant to resource-limited settings.

Consistent with studies from South Africa, Kenya, and Nigeria, we identified *Staphylococcus aureus*—both MSSA and MRSA—as the leading pathogen associated with septic embolic disease in children (26–29). Similar patterns have been reported in cohorts of African children with osteomyelitis, skin infections, and septic thrombophlebitis, where *Staphylococcus aureus* accounts for 40%–70% of cases (30, 31). In our cohort, staphylococcal species represented the majority of culture-confirmed infections, suggesting that the microbiological drivers of SPE in Somali children mirror those described elsewhere on the continent. The presence of coagulase-negative staphylococci (CoNS) in several cases aligns with observations from African pediatric series, where CoNS may act as true pathogens in the context of venous thrombosis or indwelling devices (32).

The clinical presentation in our cohort — with high fever, respiratory symptoms, myalgia, and elevated inflammatory markers — is similar to descriptions from pediatric SPE cases reported in Egypt (33, 34). However, the complete absence of documented hypotension at admission contrasts with reports from tertiary centers in South Africa and Rwanda, where 10%–20% of children with severe staphylococcal sepsis present with hemodynamic compromise (35, 36). This could reflect earlier presentation, different clinician thresholds for documenting shock, or limitations in vital-sign recording.

Radiologically, the predominance of peripheral nodules, cavitation, and pleural complications aligns with patterns described across African pediatric SPE series (37–39). Empyema was less common in our cohort (7%) than in some South African studies, where up to 20%–30% of children with SPE develop pleural involvement requiring drainage (40). Variability in imaging availability—particularly limited access to CT in many Somali hospitals—may contribute to under-recognition of subtle pulmonary lesions and thrombotic phenomena.

A striking feature of our cohort is the high rate of anticoagulation use (93%), exceeding that reported in Nigerian pediatric venous thromboembolism studies, where use typically ranges from 40% to 70%, reflecting variability in local guidelines, resource availability (41, 42). The strong anticoagulation trend in our hospital likely reflects awareness of extensive septic thrombophlebitis as a central pathogenic process and the clinical emphasis on preventing embolic progression. Nevertheless, optimal anticoagulation strategies in children with SPE remain debated in African and international literature (38, 43, 44).

Another notable difference concerns ICU admission patterns. In our cohort, one-third (33%) of children required intensive care, which is broadly comparable to the 35% ICU admission reported in the Egyptian pediatric SPE series by Elmeazawy and El Amrousy (2023) (2). Limited pediatric data from sub-Saharan Africa preclude reliable comparison, as published Kenyan and Nigerian series do not report ICU admission rates for SPE (45). The strong effect sizes observed for younger age, higher WBC, and markedly prolonged hospital stay in ICU patients reinforce the severity of illness observed in this subgroup. The absence of mortality in our series is encouraging, though may reflect the small sample size; African studies of pediatric SPE report mortality rates ranging from 5% to 15% (2, 10, 27, 34).

Overall, our findings contribute valuable data on SPE in Somali children, a population for whom published evidence remains scarce. The strong association of SPE with *Staphylococcus aureus* infection, soft-tissue sepsis, and thrombotic complications mirrors patterns described across sub-Saharan Africa. However, differences in ICU use, anticoagulation practices, and clinical presentation highlight the need for context-specific diagnostic and management pathways.

Future research should aim to include multicenter Somali cohorts, incorporate advanced imaging modalities where feasible, and evaluate standardized approaches to antimicrobial and anticoagulation therapy in children with SPE. Improved microbiology capacity—including rapid diagnostics—may further enhance the accuracy of pathogen detection and guide stewardship efforts.

The high proportion of negative cultures (33.3%) represents an important diagnostic limitation. This may be explained by prior empirical antibiotic administration before culture sampling, which is common in resource-limited settings and significantly reduces microbiological yield. In addition, limitations in laboratory capacity, including restricted access to advanced culture techniques and molecular diagnostics, may have contributed to under-detection of pathogens. Additionally, the unavailability of advanced biomarkers such as procalcitonin and molecular diagnostics (e.g., PCR) may have further limited diagnostic precision. It is also possible that infections were caused by fastidious or atypical organisms not detectable by standard methods. This limitation has direct implications for antimicrobial stewardship, as the absence of microbiological confirmation necessitates reliance on broad-spectrum empirical therapy, limiting opportunities for targeted treatment and potentially increasing the risk of antimicrobial resistance.

## Conclusion

This study highlights septic pulmonary embolism in Somali children as a largely staphylococcal infection linked to skin, soft-tissue, and trauma-related sepsis. Despite limited diagnostic resources, characteristic imaging findings and strong inflammatory responses were consistently detected. ICU patients were younger, more inflamed, and stayed longer, but encouragingly no deaths occurred, suggesting good outcomes with timely antibiotics, anticoagulation, and supportive care. These findings provide much-needed data from a region where pediatric SPE is rarely reported.

These findings highlight the importance of early recognition and combined clinical–radiological assessment in guiding management of SPE in resource-limited settings. However, the results should be interpreted as hypothesis-generating and require validation in larger prospective studies.

### Strengths and limitations

This study provides the first pediatric description of septic pulmonary embolism in Somalia, offering detailed clinical, laboratory, radiological, and microbiological data despite a resource-limited setting. The use of effect size measures and a structured comparison of ICU versus non-ICU patients adds depth to the analysis.

Given the small sample size and limited number of outcome events, this study lacks sufficient statistical power to support definitive conclusions. Due to this limitation, more advanced statistical analyses such as multivariable regression or Cox proportional hazards modeling were not feasible, further restricting the ability to identify independent predictors of outcomes and reinforcing the exploratory nature of this study. Therefore, the findings should be interpreted as hypothesis-generating rather than confirmatory, and larger prospective multicenter studies are required to validate these observations.

The retrospective design introduces inherent risks of selection and information bias. Patient inclusion depended on available clinical records and diagnostic documentation, which may not fully represent all cases during the study period. Additionally, variability in documentation quality may have resulted in incomplete or inconsistent data capture. These limitations may influence the observed associations and restrict the generalizability of the findings**.**

However, the small sample size, retrospective design, and limited access to advanced imaging and microbiology reduce generalizability and may underestimate thrombotic and infectious burdens. A high proportion of negative cultures and the absence of mortality also restricted multivariable analysis and limited the ability to identify independent predictors of severity.

## Data Availability

The raw data supporting the conclusions of this article will be made available by the authors, without undue reservation.
